# Does Electron Capture Dissociation Cleave Protein Disulfide Bonds?

**DOI:** 10.1002/open.201200038

**Published:** 2012-11-30

**Authors:** Barbara Ganisl, Kathrin Breuker

**Affiliations:** a Institute for Organic Chemistry and Center for Molecular Biosciences Innsbruck (CMBI), University of InnsbruckInnrain 80–82, 6020 Innsbruck (Austria) E-mail: kathrin.breuker@uibk.ac.at

**Keywords:** disulfide bonds, electron capture dissociation, mass spectrometry, proteins

## Abstract

Peptide and protein characterization by mass spectrometry (MS) relies on their dissociation in the gas phase into specific fragments whose mass values can be aligned as ‘mass ladders’ to provide sequence information and to localize possible posttranslational modifications. The most common dissociation method involves slow heating of even-electron (*M*+*n* H)^*n*+^ ions from electrospray ionization by energetic collisions with inert gas, and cleavage of amide backbone bonds. More recently, dissociation methods based on electron capture or transfer were found to provide far more extensive sequence coverage through unselective cleavage of backbone N–Cα bonds. As another important feature of electron capture dissociation (ECD) and electron transfer dissociation (ETD), their unique unimolecular radical ion chemistry generally preserves labile posttranslational modifications such as glycosylation and phosphorylation. Moreover, it was postulated that disulfide bond cleavage is preferred over backbone cleavage, and that capture of a single electron can break both a backbone and a disulfide bond, or even two disulfide bonds between two peptide chains. However, the proposal of preferential disulfide bond cleavage in ECD or ETD has recently been debated. The experimental data presented here reveal that the mechanism of protein disulfide bond cleavage is much more intricate than previously anticipated.

## Introduction

Over the past two decades, mass spectrometry (MS) became central to the identification and characterization of proteins and their posttranslational modifications. Among the latter, disulfide bonds are of particular importance as their covalent R^1^–S–S–R^2^ linkages can substantially stabilize a protein’s fold against denaturation.^1^ However, disulfide bonds can also seriously complicate sequence analysis using MS by preventing fragment ion separation in protein dissociation experiments.^2^

**Scheme 1:**
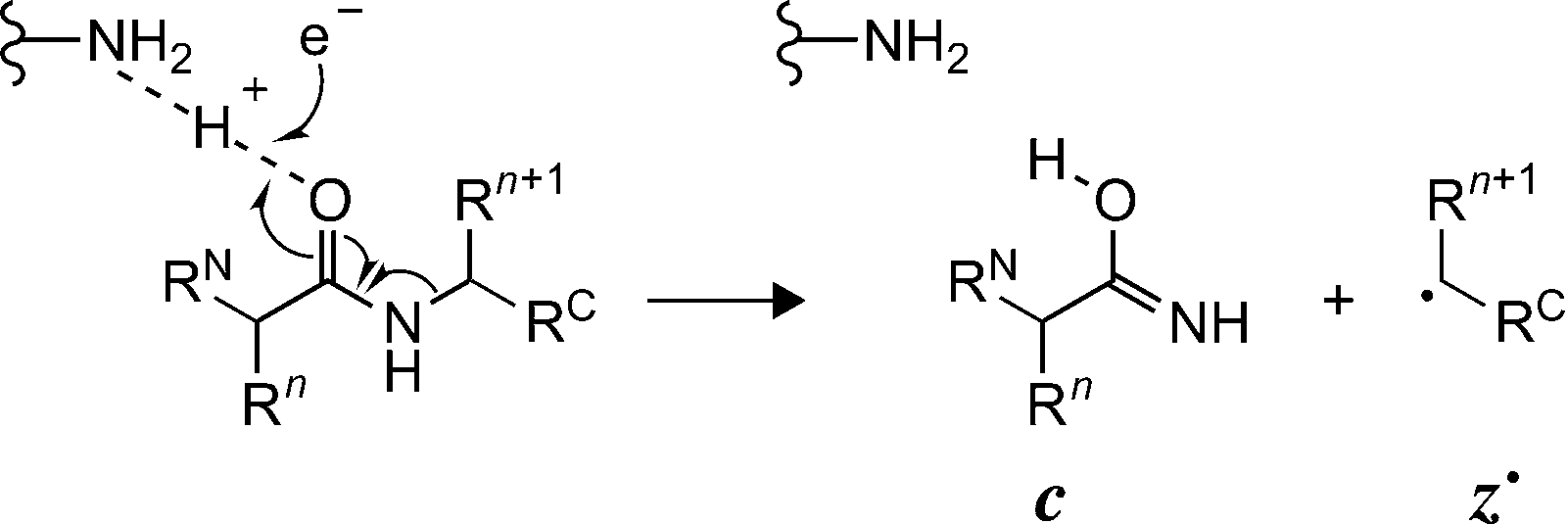
Proposed mechanism for *c* and *z*^.^ fragment formation in ECD.^3^

Electron capture dissociation (ECD), introduced by McLafferty and coworkers in 1998,^3^ revolutionized peptide and protein MS by providing far more extensive sequence coverage^4^ than conventional dissociation methods based on ‘slow ion heating’,^5^ such as collisionally activated dissociation (CAD). A year later, in 1999, the McLafferty group published a study in which it was concluded that disulfide bond cleavage is preferred over backbone cleavage into *c* and complementary *z*^.^ fragments (Scheme 1) in ECD of peptides and proteins.^6^ The proposed mechanism for disulfide bond cleavage involves a mobile H^.^ atom and its transfer to the disulfide bond. Although this study is highly cited (320 times, as of November 2012), and the proposed mechanism debated, little experimental data has since provided conclusive evidence for preferential cleavage of disulfide bonds in ECD or its closely related descendant, electron transfer dissociation (ETD).^7^ Here, we reinvestigate by experiment the question of whether or not, and how, protein disulfide bonds are cleaved during ECD. Our comprehensive study has important implications for protein dissociation experiments using ECD or ETD, and their as yet debated mechanisms.

## Results

This study was sparked by an observation that appeared to contradict the hypothesis of preferential disulfide bond cleavage in ECD. Specifically, in ECD of the protein ecotin, we found *c* and *z*^.^ fragments from backbone cleavage exclusively in regions that are not bridged by its disulfide bond. To further investigate this phenomenon, we have studied the disulfide-bonded proteins, trypsin inhibitor, insulin, aprotinin, and the peptides K8- and R8-vasopressin by ECD, with and without vibrational excitation by collisions and infrared (IR) laser heating before and after electron capture, respectively.

### Ecotin

**Figure 1:**
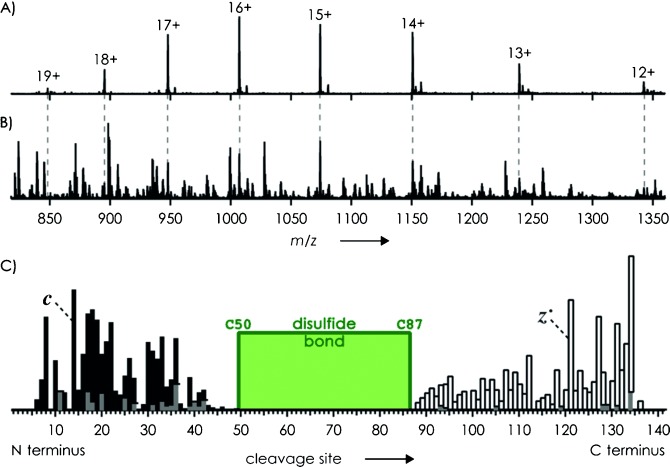
A) ESI spectrum of ecotin (1 μm in 50:50:1 H_2_O/CH_3_OH/CH_3_COOH, pH 2.5) and B) spectrum after exposure of all (*M*+*n* H)^*n*+^ ions to low-energy electrons (<1 eV). C) Relative abundances of *c* (▪), *z*^.^ (□), and *a*^.^ and *y* (▪) fragments from ECD; green box indicates region bridged by the disulfide bond.

Figure 1 A shows an electrospray ionization (ESI) spectrum of ecotin, a 142-residue protein with a single disulfide bond between C50 and C87 (see Experimental Section). ECD of all (*M*+*n* H)^*n*+^ ions (*n*=12–19) gave extensive fragmentation (Figure 1 B); site-specific, relative abundances of *c* and *z*^.^ fragments, along with minor *a*^.^ and *y* products,^6^ are displayed in Figure 1 C. Surprisingly, not a single fragment from backbone cleavage in the disulfide-bridged region (sites 50–86) was found in the ECD spectrum, suggesting that the disulfide bond was still intact and prevented separation of fragments from backbone cleavage in the disulfide-bridged region. Further, loss of S, ^.^SH, or SH_2_ from (*M*+*n* H)^(*n*−1)+.^ ions as possible indicators of disulfide bond cleavage was not observed in the ECD spectrum in Figure 1 B. However, the N- and C-terminal regions in Figure 1 C are dominated by *c* and *z*^.^ fragments, respectively, indicating substantial secondary dissociation as a result of secondary electron capture predominantly by larger fragments, which generally carry more charge than smaller fragments and have correspondingly higher cross sections for electron capture.^8^ While the absence of fragments from cleavage at sites 50–86 suggested that the disulfide bond was preserved during ECD, we could not rule out the possibility that the observed fragmentation pattern was merely a result of secondary electron capture, and therefore decided to study other proteins with different disulfide bond connectivity.

Along with the *c* and *z*^.^ fragments found in the ECD spectra reported below for trypsin inhibitor, insulin, aprotinin and vasopressin, we also observed *a*^.^ and *y* fragments. However, as also found for peptides,^9^ these were generally from the same backbone cleavage sites as the *c*, *z*^.^ fragments and of far lower abundance, and not included in the analyses. *M* used in, for example, (*M*+*n* H)^*n*+^ generally refers to the peptide or protein with fully intact disulfide bonds. None of the proteins or peptides studied here comprises cysteine residues that are not involved in disulfide bonding. All peptides and proteins were electrosprayed from 1 μm solutions in 50:50:1 water/methanol/acetic acid at pH 2.5 to promote their unfolding in solution and the formation of highly charged (*M*+*n* H)^*n*+^ ions during ESI.

### Trypsin Inhibitor

**Figure 2:**
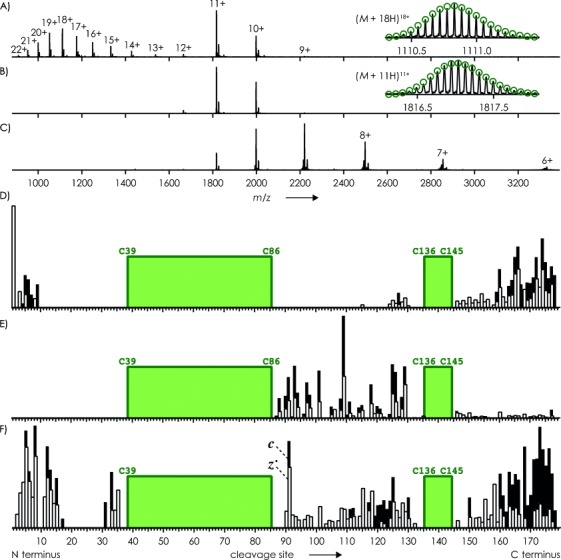
A) ESI spectrum of trypsin inhibitor (1 μm in 50:50:1 H_2_O/CH_3_OH/CH_3_COOH, pH 2.5, B) spectrum after isolation of (*M*+*n* H)^*n*+^ ions with *n*=9–12, and C) spectrum after exposure of isolated ions to low-energy electrons. Relative abundances of *c* (▪) and *z*^.^ (□) fragments from ECD of isolated ions with prior collisional activation using D) 5 V and E) 10 V potentials, and F) from IR laser heating of (*M*+11 H)^10+.^ ions formed by electron capture of (*M*+11 H)^11+^ ions. Insets in A) and B) show measured (lines) and calculated (green circles) isotopic profiles for (*M*+18 H)^18+^ and (*M*+11 H)^11+^ ions with both disulfide bonds intact; green boxes indicate regions bridged by disulfide bonds.

We next studied trypsin inhibitor from soybean (180 residues, see Experimental Section) which comprises two non-overlapping disulfide bonds (C39/C86 and C136/C145) and three unbridged regions. The ESI spectrum of trypsin inhibitor (Figure 2 A) shows a bimodal charge distribution, suggesting the presence of both folded and unfolded protein in solution.^10^ However, the isotopic profiles of all (*M*+*n* H)^*n*+^ ions showed that both disulfide bonds were fully intact (Figure 2 A and B). Isolation of the ions with *n*=9–12, which possibly originated from folded protein in solution, gave the spectrum shown in Figure 2 B; ECD of these ions after gentle collisional activation (5 V potential, corresponding to 45–60 eV laboratory frame energy) gave the spectrum in Figure 2 C. No *c* and *z*^.^ fragments from backbone cleavage at sites 10–114 were observed (Figure 1 D), suggesting that this region of the protein was still folded in the gas phase.^11^ More rigorous collisional activation (10 V potential, corresponding to 90–120 eV) of the *n*=9–12 ions before ECD gave *c* and *z*^.^ fragments from backbone cleavage in regions 87–135 and 146–179 (Figure 2 E), indicating significant unfolding of the gas-phase ions as a result of vibrational excitation. Nevertheless, fragment ions from backbone cleavage in the regions bridged by disulfide bonds were not observed at either 5 or 10 V, suggesting that both disulfide bonds were still intact, even after capture of up to four electrons (Figure 2 C).

To address the question whether a single electron can cause both disulfide and backbone bond cleavage as postulated previously,^6^ we isolated (*M*+11 H)^10+.^ ions from single-electron capture of (*M*+11 H)^11+^ ions of trypsin inhibitor, and exposed them to IR laser radiation (180 ms, 25 % power) to separate any *c* and *z*^.^ fragments that were still held together by noncovalent bonding. Again, no fragments from backbone cleavage in the regions bridged by disulfide bonds were observed, although IR laser heating separated numerous *c* and *z*^.^ fragments from cleavage in all unbridged regions (Figure 2 F).

**Figure 3:**
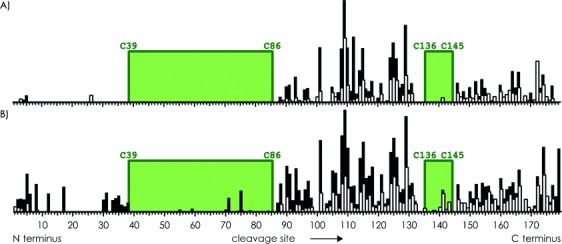
Relative abundances of *c* (▪) and *z*^.^ (□) fragments from ECD of isolated (*M*+*n* H)^*n*+^ ions of trypsin inhibitor with *n*=16–20 and prior collisional activation using A) 2.5 V and B) 5 V potentials. Green boxes indicate regions bridged by disulfide bonds.

ECD of more highly charged (*M*+*n* H)^*n*+^ ions of trypsin inhibitor (*n*=16–20), which possibly originated from unfolded protein in solution, gave rather unselective backbone cleavage in the unbridged center and C-terminal regions (Figure 3). However, only a single fragment, *z*_39_^.^, resulted from backbone cleavage in a disulfide-bridged region (Figure 3 A) when gentle collisional activation (2.5 V, corresponding to 40–50 eV) was used for unfolding before ECD. At twice the energy (80–100 eV), the number of backbone cleavages in the disulfide-bridged regions increased to 14, but most of the *c* and *z*^.^ fragments were still from cleavage in the unbridged regions (Figure 3 B).

Irrespective of precursor ion charge, fragment ions from cleavage in the N-terminal region (sites 1–38) of trypsin inhibitor were generally scarce (Figure 2 and 3), consistent with its relatively small number of basic residues available for protonation and electron capture (N terminus, R30, R38). Loss of S, ^.^SH, or SH_2_ from (*M*+*n* H)^(*n*−1)+.^ ions of trypsin inhibitor was not observed in any of the spectra in Figure 2 and 3.

### Insulin

**Figure 4:**
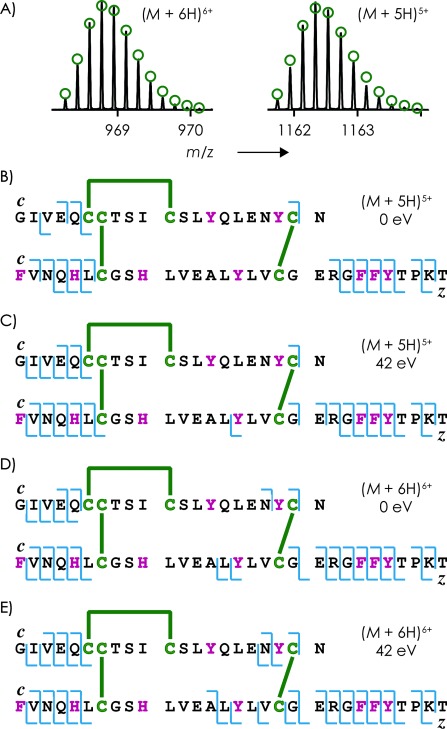
A) Measured (lines) and calculated (green circles) isotopic profiles of (*M*+6 H)^6+^ and (*M*+5 H)^5+^ ions of insulin (1 μm solution in 50:50:1 H_2_O/CH_3_OH/CH_3_COOH, pH 2.5). B–E) Schematic representation of *c* and *z*^.^ fragments (blue lines) observed in ECD of (*M*+5 H)^5+^ and (*M*+6 H)^6+^ ions without and with 42 eV collisional activation as indicated; disulfide bonds are indicated as green lines, and aromatic residues (F, H, Y) are shown in purple.

Human insulin consists of two separate peptide chains (chain A and B; see Experimental Section), and has three disulfide bonds, two between chains A and B (C7 of A with C7 of B, and C20 of A with C19 of B), and one between C6 and C11 of chain A. ESI of insulin produced mostly (*M*+5 H)^5+^ and (*M*+6 H)^6+^ ions, whose isotopic profiles showed that all three disulfide bonds were fully intact (Figure 4 A).

ECD of the (*M*+5 H)^5+^ ions without prior collisional activation, and under conditions of capture of up to three electrons, gave *c* and *z*^.^ fragments from backbone cleavage only in regions not bridged by disulfide bonds (Figure 4 B), and, in contrast to the 1999 study by McLafferty and coworkers,^6^ we observed no signals originating from separated A or B chains. With collisional activation (42 eV) before ECD, a small signal corresponding to *z*_15_^.^ from backbone cleavage in a disulfide-bridged region appeared (Figure 4 C), along with a small signal corresponding to separated B chain; signals from the less basic A chain were not observed.

ECD of the (*M*+6 H)^6+^ ions without collisional activation gave one *c* and two *z*^.^ fragments from backbone cleavage in disulfide-bridged regions (Figure 4 D), and small yields of separated chains A and B (3 %, compared to 19 % fragments from backbone cleavage and 78 % reduced molecular ions). Collisional activation (42 eV) before ECD increased the number of *c* and *z*^.^ fragments from backbone cleavage in disulfide-bridged regions to two and five, respectively, without significantly changing the overall fragmentation pattern (Figure 4 E) or the yield of separated A and B chains (5 %, compared to 45 % fragments from backbone cleavage and 50 % reduced molecular ions).

In ECD of both (*M*+5 H)^5+^ and (*M*+6 H)^6+^ ions of insulin, all *c* and *z*^.^ fragment ions from cleavage in disulfide-bridged regions were from sites next to or close to tyrosine residues (Figure 4 C–E). Moreover, all ECD spectra of insulin showed ∼1 % loss of ^.^SH from (*M*+*n* H)^(*n*−1)+.^ ions.

### Aprotinin

ESI of aprotinin, a small protein (58 residues; see Experimental Section) that is also known as bovine pancreatic trypsin inhibitor (BPTI), gave mostly (*M*+6 H)^6+^ and (*M*+7 H)^7+^ ions. Initial ECD experiments using (*M*+6 H)^6+^ ions without prior collisional activation showed some *c* and *z*^.^ fragments from backbone cleavage in regions bridged by disulfide bonds, in unexpected contrast to the data for ecotin, trypsin inhibitor, and insulin.

**Figure 5:**
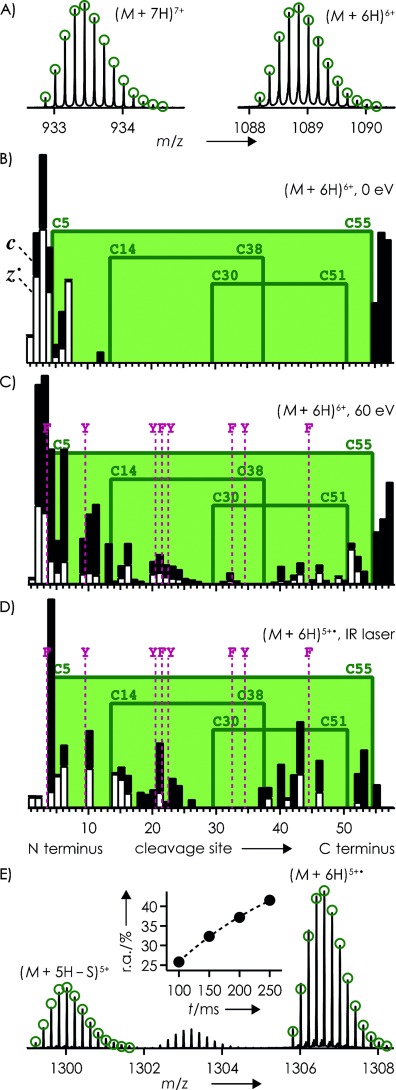
A) Measured (lines) and calculated (green circles) isotopic profiles of (*M*+7 H)^7+^ and (*M*+6 H)^6+^ ions of oxidized aprotinin (1 μm in 50:50:1 H_2_O/CH_3_OH/CH_3_COOH, pH 2.5) from ESI. Relative abundances of *c* (▪) and *z*^.^ (□) fragments from ECD of (*M*+6 H)^6+^ ions B) without and C) with 60 eV collisional activation prior to ECD, and D) from IR laser heating of (*M*+6 H)^5+.^ ions; green boxes indicate regions bridged by disulfide bonds, and locations of aromatic residues are indicated by purple lines. E) *m/z* region illustrating ^.^SH loss from IR laser heating (200 ms, 35 %) of (*M*+6 H)^5+.^ ions; green circles indicate calculated isotopic profiles for (*M*+6 H)^5+.^ and (*M*+5H−S)^5+^; inset shows abundance of (*M*+5H−S)^5+^ relative to (*M*+6 H)^5+.^ versus IR laser irradiation time.

To exclude the possibility that incomplete disulfide bond formation was responsible for this result, the stock solution of aprotinin (16 μm) was incubated with H_2_O_2_ (6 % v/v) for ∼45 min. This treatment resulted in complete oxidation of M52, and the isotopic profiles of (*M*+6 H)^6+^ and (*M*+7 H)^7+^ ions showed that all three disulfide bonds were fully intact (C5/C55, C14/C38, C30/C51; Figure 5 A).

Without prior collisional activation and under conditions of capture of up to three electrons, ECD of the (*M*+6 H)^6+^ ions of aprotinin gave only a few fragments from backbone cleavage at sites 1–7 and 12 (Figure 5 B). Using a 10 V potential, corresponding to 60 eV laboratory frame energy, for collisional activation before ECD, under otherwise unchanged experimental conditions, *c* and *z*^.^ fragments at 43 out of 57 possible sites were observed, including sites 31, 32, and 33, which are bridged by a total of three disulfide bonds (Figure 5 C). The ECD spectra in Figure 5 B and 5 c showed 9 % and 11 % loss of ^.^SH from (*M*+6 H)^5+.^ ions, respectively.

IR laser heating (200 ms, 35 %) of (*M*+6 H)^5+.^ ions of aprotinin from single-electron capture by (*M*+6 H)^6+^ ions also gave *c* and *z*^.^ fragments from backbone cleavage in all regions of the protein (Figure 5 D). Compared with the fragmentation pattern from ECD of (*M*+6 H)^6+^ ions with 60 eV collisional activation before ECD (Figure 5 C), relative *c* and *z*^.^ fragment ion abundances from IR laser heating were somewhat higher at cleavage sites next to residues with aromatic side chains (Figure 5 D), and loss of ^.^SH from (*M*+6 H)^5+.^ ions increased from 26 to 42 %, when increasing the laser pulse length from 100 to 250 ms at 35 % power (Figure 5 E).

**Figure 6:**
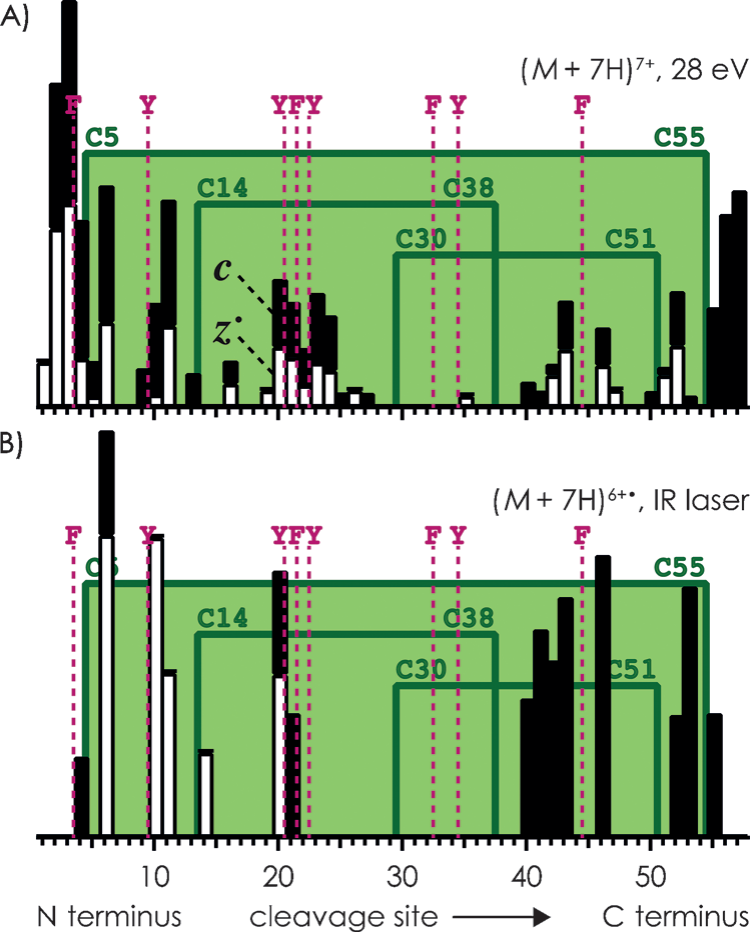
Relative abundances of *c* (▪) and *z*^.^ (□) fragments A) from ECD of (*M*+7 H)^7+^ ions of aprotinin with 28 eV collisional activation prior to ECD, and B) from IR laser heating of (*M*+7 H)^6+.^ ions.

ECD of the (*M*+7 H)^7+^ ions of aprotinin with prior collisional activation, using only 28 eV laboratory frame energy, gave a fragmentation pattern similar to that from ECD of (*M*+6 H)^6+^ ions at 60 eV (cf. Figures 6 A and 5 C). IR laser heating (100 ms, 35 %) of (*M*+7 H)^6+.^ from single-electron capture by (*M*+7 H)^7+^ ions separated *c* and *z*^.^ fragment ions from backbone cleavage in all regions except for that bridged by three disulfide bonds (Figure 6 B). Similar to IR laser heating of the (*M*+6 H)^5+.^ ions, increasing the laser pulse length in steps of 50 ms, up to a total of 250 ms, increased the number of products corresponding to loss of small neutral species (NH_3_, H_2_O, ^.^SH) from (*M*+7 H)^6+.^ and larger fragment ions. Nevertheless, a major product from IR laser heating of the (*M*+7 H)^6+.^ ions of aprotinin at any laser pulse length corresponded to loss of ^.^SH, producing even-electron (*M*+6 H−S)^6+^ ions.

### Vasopressin

**Figure 7:**
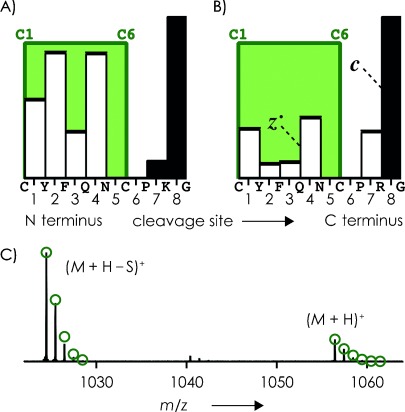
Relative abundances of *c* (▪) and *z*^.^ (□) fragments from ECD of (*M*+2 H)^2+^ ions of A) K8-vasopressin and B) R8-vasopressin (both 1 μm in 50:50:1 H_2_O/CH_3_OH/CH_3_COOH, pH 2.5) without prior collisional activation; green boxes indicate regions bridged by disulfide bonds. C) *m/z* region of ECD spectrum of (*M*+2 H)^2+^ ions of K8-vasopressin illustrating H^.^ and ^.^SH loss from (*M*+2 H)^+⋅^ ions; green circles indicate calculated isotopic profiles for (*M*+H)^+^ and (*M*+H−S)^+^.

ECD of the (*M*+2 H)^2+^ ions of the small peptides K8- and R8-vasopressin (see Experimental Section), both of which have a disulfide bond between C1 and C6, gave *c* and *z*^.^ fragments from backbone cleavage at nearly all possible sites even without prior collisional activation (Figure 7 A and 7 B); 6 eV activation prior to ECD gave virtually the same spectra and *c*, *z*^.^ fragmentation patterns. As illustrated in Figure 7 C for K8-vasopressin, a major product in ECD of the (*M*+2 H)^2+^ ions of vasopressin, with and without prior collisional activation, resulted from loss of ^.^SH from (*M*+2 H)^+.^, and all (M+2 H)^+.^ ions showed loss of H^.^.

## Discussion

Each of the two non-overlapping disulfide bonds of trypsin inhibitor (C39/C86 and C136/C145) has a basic residue as direct sequence neighbor of one of the cysteines involved in disulfide bonding (R38 and C39, K144 and C145). According to theoretical predictions,^12^ the positive charge on the neighboring basic residues should either allow direct electron attachment into the disulfide σ* orbitals, or electron attachment into Rydberg orbitals of the positively charged sites of R38 and K144, followed by fast (∼10^−6^ s) relaxation into lower-energy Rydberg levels suitable for electron transfer to disulfide σ* orbitals, with either scenario resulting in disulfide bond cleavage. However, even under conditions of capture of up to four electrons and vibrational excitation (up to 120 eV laboratory frame energy) for ion unfolding before electron capture, we observed no products indicating disulfide bond cleavage in ECD of (*M*+*n* H)^*n*+^ ions of trypsin inhibitor with *n*=9–12 (Figure 2 D and 2 E).

Moreover, gentle IR laser heating of (*M*+11 H)^10+.^ ions from single-electron capture of (*M*+11 H)^11+^ ions of trypsin inhibitor separated *c* and *z*^.^ fragments from cleavage at nearly all sites except those bridged by disulfide bonds (Figure 2 F). This data shows that capture of a single electron by (*M*+11 H)^11+^ ions of trypsin inhibitor does not bring about both disulfide and backbone bond cleavage. Instead, the data indicates virtually unselective backbone cleavage into *c* and *z*^.^ fragments while fully preserving the C39/C86 and C136/C145 disulfide bonds.

A similar fragmentation pattern was observed in ECD of the more highly charged (*M*+*n* H)^*n*+^ ions of trypsin inhibitor with *n*=16–20 under conditions of gentle collisional activation before ECD (Figure 3 A). Only with more rigorous collisional activation before ECD did the number of separated *c* and *z*^.^ fragments from backbone cleavage in disulfide-bridged regions increase significantly (Figure 3 B), but not nearly to the extent observed in unbridged regions. Apparently, collisional activation *before* ECD, and not electron capture, caused disulfide bond cleavage in some of the more highly charged (*M*+*n* H)^*n*+^ ions of trypsin inhibitor. In support of this hypothesis, are data from ETD of monoclonal antibodies by Tsybin and coworkers, who observed *c* and *z^.^* fragments mostly from backbone cleavage in regions not bridged by disulfide bonds.^13^

Insulin, which has three disulfide bonds but consists of two separate peptide chains, also shows evidence for disulfide bond cleavage as a result of vibrational excitation; ECD of its (*M*+5 H)^5+^ ions under conditions of capture of up to three electrons and without prior collisional activation gave no *c* and *z*^.^ fragments from backbone cleavage in regions bridged by disulfide bonds (Figure 4 B), and no separated A or B chains, whereas collisional activation before ECD gave two products indicating disulfide bond cleavage (Figure 4 C). Likewise, the number of *c*, *z*^.^ fragment ions from cleavage in disulfide-bridged regions increased with prior collisional activation of the (*M*+6 H)^6+^ ions of insulin (Figure 4 D and 4 E). A similar effect of vibrational excitation on disulfide bond cleavage was observed by McLuckey and coworkers, who found *c* and *z*^.^ fragments from backbone cleavage in regions bridged by disulfide bonds and separated A or B chains in ETD of insulin (*M*+*n* H)^*n*+^ ions with *n*=3–6 only with collisional activation of the (*M*+*n* H)^(*n*−1)+.^ ions.^14^

Like insulin, aprotinin has three disulfide bonds, and also comprises a similar number of residues (insulin: 51, and aprotinin: 58), but aprotinin consists of a single peptide chain. The three disulfide bonds (C5/C55, C14/C38, C30/C51) divide the protein backbone into regions bridged by up to three disulfide bonds; neighboring basic residues are K15 (C14), R39 (C38), and R53 (C51, C55). Without collisional activation and under conditions of capture of up to three electrons, ECD of the (*M*+6 H)^6+^ ions gave *c* and *z*^.^ fragments from backbone cleavage in unbridged and singly, but not doubly or triply, bridged regions (Figure 5 B). With collisional activation before ECD, *c* and *z*^.^ fragments from backbone cleavage in all regions were observed (Figure 5 C). Disulfide bond cleavage in the (*M*+7 H)^7+^ ions (Figure 5 D) required only about half the energy (28 eV) than that for the (*M*+6 H)^6+^ ions (60 eV), consistent with increasing Coulomb repulsion decreasing overall ion stability.

The data discussed so far show that collisional activation *before* ECD generally increases the number of *c* and *z*^.^ fragments from backbone cleavage in disulfide-bridged regions, strongly suggesting that vibrational excitation can cleave protein disulfide bonds, at least to some extent. This finding is consistent with recent studies reporting disulfide bond cleavage, along with backbone cleavage into *b* and *y* fragments, in collisionally activated dissociation of even-electron (*M*+*n* H)^*n*+^ protein^15^ and peptide^16^ ions.

However, mere vibrational excitation can obviously not account for the observed ^.^SH losses and apparently coinciding disulfide bond cleavages in ECD of insulin (Figure 4 D) and aprotinin (Figure 5 B) *without* prior collisional activation or subsequent IR laser heating. This data instead suggests that radical ion chemistry was involved in at least some of the disulfide bond cleavages. Concurrent disulfide bond cleavage and ^.^SH loss was also observed in ETD of somatostatin, a 14 residue peptide with a disulfide bond between C3 and C14,16a and other small peptides comprising a single disulfide bond.^17^

If radical reactions can cleave disulfide bonds in ECD, are these affected by vibrational excitation? It was shown that ion heating before or after ECD can break noncovalent bonds in protein ions with more compact structures that could otherwise prevent separation of *c* and *z*^.^ fragments from backbone cleavage,^4^, 11a, c, 18 consistent with the data for trypsin inhibitor (Figure 2 D–F). By contrast, heating of protein ions with elongated structures, for example, the (*M*+13 H)^13+^ ions of ubiquitin,^19^ showed no increase in yields of the even-electron *c* ions with increasing ion temperature from 25 to 125 °C,^20^ which corroborates the postulate of nonergodic dissociation in ECD.^3^ Yields of the complementary, radical *z*^.^ ions (Scheme 1), however, were found to actually decrease with increasing ion temperature, which was attributed to their lower stability and higher reactivity.11a, 20–21 In other words, primary backbone cleavage into *c* and *z*^.^ fragments, which is not affected by vibrational excitation,^20^ can be followed by secondary reactions that involve the radical *z*^.^ ions. Evidence for secondary reactions of radical *z*^.^ ions was also found in ECD of linear^21^ and cyclic peptides.^22^ These radical reactions are generally fast, proceeding on a sub-millisecond timescale,^23^ and—in striking contrast to primary backbone cleavage into *c* and *z*^.^ fragments—become more efficient with increasing ion temperature.11a The increase in ^.^SH loss from (*M*+6 H)^5+.^ ions of aprotinin with increasing vibrational excitation (Figure 5 E) strongly suggests that the radical pathway resulting in disulfide bond cleavage involves secondary radical reactions of the *z*^.^ ions from primary backbone cleavage. Note that the energy deposited per residue in IR laser heating of (*M*+6 H)^5+.^ ions of aprotinin (Figure 5 E) was 2.4–6.1 times higher than that used for IR laser heating of (*M*+11 H)^10+.^ ions of trypsin inhibitor (Figure 2 F). This qualitatively agrees with lower energy requirements for breaking of noncovalent bonds in trypsin inhibitor to separate *c* and *z*^.^ fragments, and higher energy requirements for facilitating secondary radical reactions of *z*^.^ ions—which involves breaking of covalent bonds—in aprotinin.

Secondary radical reactions can be part of radical ′cascade reactions′,^22^, ^24^ which provides a rationale for how IR laser heating of the (*M*+6 H)^5+.^ ions of aprotinin, formed by capture of a single electron, cleaved up to three disulfide bonds and the protein backbone (Figure 5 D). Possibly these cascade reactions are preferentially interrupted at sites of higher radical stability^25^ such as residues with aromatic side chains, thereby increasing the relative number of *c* and *z*^.^ fragments from backbone cleavage next to tyrosine and phenylalanine residues (Figures 4–6).

So why do we find strong evidence for secondary radical reactions in ECD of insulin and aprotinin, but not for ecotin and trypsin inhibitor? A major difference between these proteins is the number of cyclic regions formed by disulfide bonds normalized to the number of residues (Table 1). Ecotin and trypsin inhibitor have rather small ′densities′ of cyclic regions, 0.007 and 0.011, respectively, meaning that the gas phase structures of their (*M*+*n* H)^*n*+^ ions, which were electrosprayed from 50:50:1 water/methanol/acetic acid solutions at pH 2.5 to promote unfolding in solution, should be rather loose, although some higher order structure is indicated in some regions of the (*M*+*n* H)^*n*+^ ions of trypsin inhibitor with *n*=9–12 (Figure 2 D–F). On the other hand, the far higher density of cyclic regions of insulin (0.039) and aprotinin (0.052) necessitates far more densely packed and compact ion structures in which radical sites from primary backbone cleavage are far closer to sites for secondary reaction, including their disulfide bonds.

**Table 1:** Density of cyclic regions in proteins studied

Protein	Residues	Cyclic regions	Density
Ecotin	142	1	0.007
Trypsin inhibitor	180	2	0.011
Insulin	21+30	2	0.039
Aprotinin	58	3	0.052
Vasopressin	9	1	0.111

To test our idea of compact ion structures facilitating secondary radical reactions in ECD, we studied the small peptides K8- and R8-vasopressin with a density of cyclic regions of 0.111 (Table 1). In strong support of our hypothesis, ECD of their (*M*+2 H)^2+^ ions gave rather unselective backbone cleavage into *c* and *z*^.^ fragments, even without prior collisional activation (Figure 7 A and 7 B). Note that N–Cα bond cleavage at site 6 does not separate *c*_6_ and the complementary *z*_3_^.^ fragments, because the side chain of P7 is covalently bound to its amide nitrogen. The only other site for which no fragment ions were observed is site 5; possibly the radical *z*_4_^.^ fragment was fully depleted by secondary reaction with the adjacent disulfide bond. Collisional activation (6 eV) prior to ECD gave virtually the same spectra and *c*, *z*^.^ fragmentation patterns, suggesting that the secondary radical reactions proceeded to completion even without vibrational excitation; as illustrated in Figure 7 C for K8-vasopressin, the (*M*+2 H)^+.^ ions from electron capture of (*M*+2 H)^2+^ ions were completely depleted by cleavage into *c* and *z*^.^ fragments, and loss of H^.^ and ^.^SH.

We have studied here cyclic peptides and proteins, but protein digestion can also give disulfide-bonded, noncyclic peptide structures.^6^, ^26^ In a study by the McLuckey group, ETD of smaller peptides from tryptic digestion (up to 14 residues in total), consisting of two chains (of up to 9 residues) connected by a single disulfide bond, gave products from both backbone and disulfide bond cleavage, whose branching ratio varied substantially with the identity and location of positively charged sites within the peptide ions.^26^ Charge location is an important factor in determining ion structure and thus ion compactness, and although the structures of the peptides studied by McLuckey and coworkers are not known, an effect of charge location on the relative extent of disulfide bond cleavage is generally consistent with our hypothesis of compact ion structures facilitating secondary radical reactions that can lead to disulfide bond cleavage. In the 1999 study by the McLafferty group, ECD of a disulfide-bonded dimer (34 residues in total) of a 17-residue peptide gave products from both backbone and disulfide bond cleavage.^6^ Among the products from backbone cleavage, *c* fragments dominated over *z*^.^ fragments, consistent with our proposal of secondary radical reactions of *z*^.^ ions leading to disulfide bond cleavage. Higher relative abundances of *c* over *z*^.^ fragments are also evident in most of the ETD spectra in reference,^26^ and the ECD spectrum of a small tryptic peptide (11 residues in total) showed only *c* but no *z*^.^ fragments, along with separated peptide chains.^27^ In multistage dissociation experiments, Wu, Hancock, Karger, and coworkers utilized this ability of ETD to separate peptide chains for the characterization of disulfide-bonded therapeutic proteins.^28^ However, in a spectrum from ECD of (*M*+10 H)^10+^ ions of a far larger peptide consisting of two chains (A: 38 and B: 48 residues) connected by a single disulfide bond, McLafferty and coworkers found very few *c* and *z*^.^ fragments, and primarily signals from reduced molecular ions and separated A and B chains.^6^ Possibly yet another mechanism for disulfide bond cleavage is operative in ECD of larger, noncyclic peptides.

## Conclusion

It turns out that the question in the title “Does electron capture dissociation cleave protein disulfide bonds?*”* cannot be answered with a simple *yes* or *no*. We found unambiguous evidence for full preservation of disulfide bonds during electron capture dissociation (ECD), even for proteins that have basic residues next to cysteines involved in disulfide bonding, and under conditions of multiple electron capture.

However, we also found unambiguous evidence that ECD can cleave disulfide bonds, although not preferentially. Instead, our data support a mechanism of electron capture at protonated sites, resulting in backbone cleavage into *c* and *z*^.^ fragment ions. This *primary* cleavage event can be followed by disulfide bond cleavage via *secondary* radical reactions, provided that the disulfide bond is in close proximity to a radical site from primary backbone cleavage. Apparently, this situation is favored in peptides or proteins with a high density of cyclic regions.

Vibrational excitation of the (*M*+*n* H)^*n*+^ ions *before* ECD can also cleave disulfide bonds, and it can promote secondary (but not primary) radical reactions in the (*M*+*n* H)^(*n*−1)+.^ ions that may lead to disulfide bond cleavage. Unintended vibrational excitation can be caused by tuning electrostatic potentials in the source region of a mass spectrometer for high ion transmission, which may explain differences in ECD fragmentation patterns found by different experimenters. Our data also show that increased ion net charge generally resulted in more disulfide bond cleavage, consistent with an increased number of possible sites for primary backbone cleavage increasing the probability for secondary reaction at disulfide bonds; similar observations were recently made by Zhang and Loo.^29^

These rather intricate effects of vibrational excitation and secondary radical reactions make it nearly impossible to predict whether or not, or to what extent, disulfide bonds of any given protein are preserved during collisionally activated dissociation (CAD), electron capture dissociation (ECD), or electron transfer dissociation (ETD). For sequencing applications, peptide or protein disulfide bonds can be fully reduced in solution prior to analysis by mass spectrometry. Elucidation of protein disulfide bond patterns by CAD, ECD or ETD, however, can be seriously complicated by complex gas phase ion chemistry.

## Experimental Section

Experiments were performed on a 7 Tesla Fourier transform ion cyclotron resonance (FT-ICR) mass spectrometer (Bruker, Austria) equipped with an electrospray ionization (ESI) source, a linear hexapole ion cell floated with argon gas for optional collisional activation, a hollow dispenser cathode for electron capture dissociation (ECD; electron energy<1 eV), and a CO_2_ laser (10.6 μm, 35 W) for IR activation in the ion cyclotron resonance (ICR) cell. Data reduction utilized SNAP2 (Bruker, Austria) and mMass software (version 3.11).^30^ Proteins and chemicals were purchased from Sigma–Aldrich (Austria). Amino acid sequences:

ecotin (142 residues)

AESVQPLEKI APYPQAEKGM KRQVIQLTPQ EDESTLKVEL

LIGQTLEVD**C** NLHRLGGKLE NKTLEGWGYD YYVFDKVSSP

VSTMMA**C**PDG KKEKKFVTAY LGDAGMLRYN SKLPIVVYTP

DNVDVKYRVW KAEEKIDNAV VR

trypsin inhibitor (180 residues)

DFVLDNEGNP LENGGTYYIL SDITAFGGIR AAPTGNER**C**P

LTVVQSRNEL DKGIGTIISS PYRIRFIAEG HPLSLKFDSF

AVIML**C**VGIP TEWSVVEDLP EGPAVKIGEN KDAMDGWFRL

ERVSDDEFNN YKLVF**C**PQQA EDDK**C**GDIGI SIDHDDGTRR

LVVSKNKPLV VQFQKLDKES

insulin (21+30 residues)

GIVEQ**CC**TSI **C**SLYQLENY**C** N (chain A) and

FVNQHL**C**GSH LVEALYLV**C**G ERGFFYTPKT (chain B)

aprotinin (58 residues)

RPDF**C**LEPPY TGP**C**KARIIR YFYNAKAGL**C** QTFVYGG**C**RA

KRNNFKSAED **C**MRT**C**GGA

K8-vasopressin (9 residues) **C**YFQN**C**PKG

R8-vasopressin (9 residues) **C**YFQN**C**PRG
